# Commentary on: Bassetti, D., Luhmann, H.J., Kirischuk, S. Presynaptic GABA_B_ receptor-mediated network excitation in the medial prefrontal cortex of Tsc2 + / − mice

**DOI:** 10.1007/s00424-021-02584-5

**Published:** 2021-05-25

**Authors:** Christian Alzheimer

**Affiliations:** Institut für Physiologie und Pathophysiologie, Friedrich-Alexander-Universität Erlangen-Nürnberg, Universitätsstr. 17, 91054 Erlangen, Germany

In a recent paper published 2020 in *Cerebral Cortex*, the group of Heiko Luhmann offered exciting new insights into the neurophysiological underpinnings of neurodevelopmental disorders, which exhibit seizures and autistic behavior as clinical hallmarks [1]. In that study, they used a mouse model of tuberous sclerosis complex (TSC), an autosomal or sporadic multisystem disorder with pronounced neuropsychiatric manifestations, including epilepsy, autism, and intellectual deficits [5]. Importantly, the human disease phenotype is well reproduced in TSC mouse models [4, 8]. On the molecular level, the TSC-associated brain symptomatology results from loss-of-function mutations in either *TSC1* or *TSC2*, which encode the mTOR complex 1 (mTORC1)-inhibiting proteins TSC1 (hamartin) and TSC2 (tuberin), respectively [5]. How aberrant mTORC1 signaling alters neuronal activity in a fashion that gives rise to seizures and impaired social behavior is not well understood.

Previous work from the group of Bernardo Sabatini in another mouse model of TSC showed that synaptic inhibition of hippocampal pyramidal cells is diminished through a postsynaptic mechanism, whereas the strength of glutamatergic neurotransmission remained unaffected, pointing to an imbalance between excitation (E) and inhibition (I) [3]. It is worth noting that disruption of the E/I balance is a core pathogenetic concept in neuropsychiatric disorders to account for both seizure proneness (neuronal hyperexcitability) and impaired information processing in the autistic brain (reduced signal-to-noise) [6, 7, 9]. While being highly instructive, the TSC model used by Sabatini’s group was somewhat limited from a translational point of view, as it was based on a conditional knockout of *TSC1* in principal forebrain neurons [3]. By contrast, all TSC patients are obligate heterozygotes, and the pathogenic mutation (in each cell of the body) involves much more often *TCS2* than *TSC1* [5].

Consequently, the Luhmann lab explored a mouse model with haploinsufficiency of *TSC2* (Tsc2^+/−^ mice) and, with their interest in autism spectrum disorders, focused their electrophysiological interrogation on acute brain slices of the medial prefrontal cortex (mPFC) [1]. Most importantly, they showed that mPFC hyperexcitability in the mutant preparation was causally linked to diminished tonic inhibition through postsynaptic GABA_B_ receptors [1]. An intriguing finding from that study was the observation that the frequency of miniature excitatory postsynaptic potentials (mEPSCs), which was increased in mutant mice when compared to their wild type (wt) controls, was counterbalanced by a delayed increase in the frequency of miniature inhibitory postsynaptic potentials (mIPSC), so that the synaptic E/I ratio, which was biased towards excitation at earlier postnatal stages, was normalized and became comparable to that of the wt preparation as the mice approached the end of the first postnatal month.

In the paper published in this issue of *Pflügers Archiv—European Journal of Physiology*, Bassetti, who was also lead author on the preceding paper, and his colleagues elaborate on their earlier findings with a particular emphasis on how pharmacological activation (with baclofen) or inhibition (with CGP55485) of presynaptic GABA_B_ receptors affects the frequency of miniature and spontaneous EPSCs and IPSCs in layer 2/3 pyramidal cells of the mPFC of Tsc2^+/−^ and wt mice 25–40 days old [2]. With respect to mEPSC frequency, reduction and increase with baclofen and CGP, respectively, were not significantly different between the two groups. By contrast, CGP failed to increase mIPSC frequency in Tsc2^+/−^, but not in wt slices, suggesting that GABA_B_ autoreceptors on GABAergic terminals are not tonically activated by ambient GABA in the mutant preparation.

In further experiments on evoked IPSCs and spontaneous EPSCs and IPSCs, the authors found that GABAergic terminals in mutant slices responded more sensitive to baclofen than those in control slices, thereby shifting the E/I ratio towards excitation in the former, whereas this parameter was hardly affected in the latter. The resulting net excitatory effect of baclofen on prefrontal neurons sends a note of caution when considering GABA_B_ receptor activation as a treatment option for neural hyperexcitability in neurodevelopmental disorders. In summary, Bassetti and colleagues have introduced a novel and unexpected pathophysiological mechanism that substantially furthers our understanding of the complex cellular and circuit alterations in a mouse model of TSC.

## References

[1] Bassetti D, Lombardi A, Kirischuk S, Luhmann HJ (2020). Haploinsufficiency of Tsc2 leads to hyperexcitability of medial prefrontal cortex via weakening of tonic GABAB receptor-mediated inhibition. Cereb Cortex.

[2] Bassetti D, Luhmann HJ, Kirischuk S (2021) Presynaptic GABA(B) receptor-mediated network excitation in the medial prefrontal cortex of Tsc2+/− mice. Pflugers Arch, this issue 10.1007/s00424-021-02576-5 PMC8302497 34279736

[3] Bateup HS, Johnson CA, Denefrio CL, Saulnier JL, Kornacker K, Sabatini BL (2013). Excitatory/inhibitory synaptic imbalance leads to hippocampal hyperexcitability in mouse models of tuberous sclerosis. Neuron.

[4] Ehninger D, Han S, Shilyansky C, Zhou Y, Li W, Kwiatkowski DJ, Ramesh V, Silva AJ (2008). Reversal of learning deficits in a Tsc2+/− mouse model of tuberous sclerosis. Nat Med.

[5] Hasbani DM, Crino PB (2018). Tuberous sclerosis complex. Handb Clin Neurol.

[6] Selimbeyoglu A, Kim CK, Inoue M, Lee SY, Hong ASO, Kauvar I, Ramakrishnan C, Fenno LE, Davidson TJ, Wright M, Deisseroth K (2017) Modulation of prefrontal cortex excitation/inhibition balance rescues social behavior in CNTNAP2-deficient mice. Sci Transl Med 9.10.1126/scitranslmed.aah6733 10.1126/scitranslmed.aah6733 PMC5723386 28768803

[7] Sohal VS, Rubenstein JLR (2019). Excitation-inhibition balance as a framework for investigating mechanisms in neuropsychiatric disorders. Mol Psychiatry.

[8] Tsai PT, Hull C, Chu Y, Greene-Colozzi E, Sadowski AR, Leech JM, Steinberg J, Crawley JN, Regehr WG, Sahin M (2012). Autistic-like behaviour and cerebellar dysfunction in Purkinje cell Tsc1 mutant mice. Nature.

[9] Yizhar O, Fenno LE, Prigge M, Schneider F, Davidson TJ, O’Shea DJ, Sohal VS, Goshen I, Finkelstein J, Paz JT, Stehfest K, Fudim R, Ramakrishnan C, Huguenard JR, Hegemann P, Deisseroth K (2011). Neocortical excitation/inhibition balance in information processing and social dysfunction. Nature.

